# The role of perivascular adipose tissue in the appearance of ectopic adipocytes in the abdominal aortic aneurysmal wall

**DOI:** 10.1080/21623945.2019.1636625

**Published:** 2019-06-28

**Authors:** Hirona Kugo, Tatsuya Moriyama, Nobuhiro Zaima

**Affiliations:** a Department of Applied Biological Chemistry, Graduate School of Agriculture, Kindai University, Nara, Japan; b Agricultural Technology and Innovation Research Institute, Kindai University, Nara, Japan

**Keywords:** Abdominal aortic aneurysm, perivascular adipose tissue, adipocyte, mesenchymal stem cells, hypoperfusion, differentiation

## Abstract

Abdominal aortic aneurysm (AAA) is a vascular disease characterized by the dilation of the abdominal aorta, resulting in a high mortality rate caused by vascular rupture. Previous studies have suggested that the abnormal appearance of adipocytes in the vascular wall is associated with the development of AAA. However, the mechanisms underlying the appearance of the ectopic adipocytes remain unknown. In this study, we showed that CD44^+^CD90^+^ MSCs express adipogenic transcription factors in the AAA wall of a hypoperfusion-induced AAA model. The number of CD44^+^CD90^+^ cells and adipocytes in the AAA wall significantly decreased in the perivascular adipose tissue (PVAT)-removed vascular wall. The AAA diameter significantly decreased in the PVAT-removed vascular wall compared with that in the vascular wall with PVAT. These data suggested that PVAT plays important roles in the differentiation of MSCs into adipocytes in response to vascular hypoperfusion. The decreased number of adipocytes in the PVAT-removed vascular wall might be associated with the decreased AAA diameter.

## Introduction

Abdominal aortic aneurysm (AAA) is characterized by the progressive dilation of the abdominal aorta, which results in a high mortality rate caused by vascular rupture. The aortic dilation progresses without any symptoms and the rupture of the AAA cannot be easily predicted. There are no medical treatments for preventing the dilation and rupture of AAAs. The few options for patients with increased risk of rupture include open surgical repair with prosthetic graft replacement or endovascular stent graft placement [1,2].

It has shown that hypoperfusion of the vascular wall due to adventitial vasa vasorum arteriosclerosis can cause AAA [3,4]. It was also reported that hypoperfusion of the vascular wall induce the abnormal appearance of vascular adipocytes, which is characteristic pathology of ruptured AAA wall that formed in a hypoperfusion-induced AAA model [5,6]. Although adipocytes in the AAA wall have not been recognized as a major pathological feature of AAA, recent studies using human AAA samples suggest the pathological importance in AAA. It is reported that the abnormal appearance of adipocytes was observed in human AAA, and the amount of triglycerides, a major component in adipocytes, in the adventitia of the vascular wall is positively correlated with the AAA diameter [5,7]. Doderer et al. reported that adipocytes were characteristically observed in the AAA wall compared with the vascular wall of popliteal artery aneurysm of which ruptures are rare[8]. Gabel *et al*. reported an adipogenic signature in molecular fingerprint for terminal AAA[9]. In addition, Krueger et al. reported the increased expression of proteolytic and inflammatory factors around the adipocytes in the adventitial layer in human AAA tissue[10]. A recent mechanical study suggested the possibility that high adipocyte content in human AAA wall is involved in the vulnerability of AAA wall[11]. These studies suggest that the abnormal appearance of adipocytes in the vascular wall is one of the important pathological events associated with the development of human AAA. However, the mechanisms underlying the abnormal appearance of adipocytes in the AAA wall remain unknown. Based on the evidence from previous human and animal studies, it has been speculated that inducing hypoperfusion in the vascular wall could cause the ectopic appearance of adipocytes. In this study, we performed a time-dependent pathological analysis of the hypoperfusion-induced vascular walls to elucidate the mechanisms underlying the abnormal appearance of adipocytes in the vascular wall.

## Results

### Time-dependent changes of vascular diameter and adipocyte number in the hypoperfusion-induced animal model

**Figure 1.**
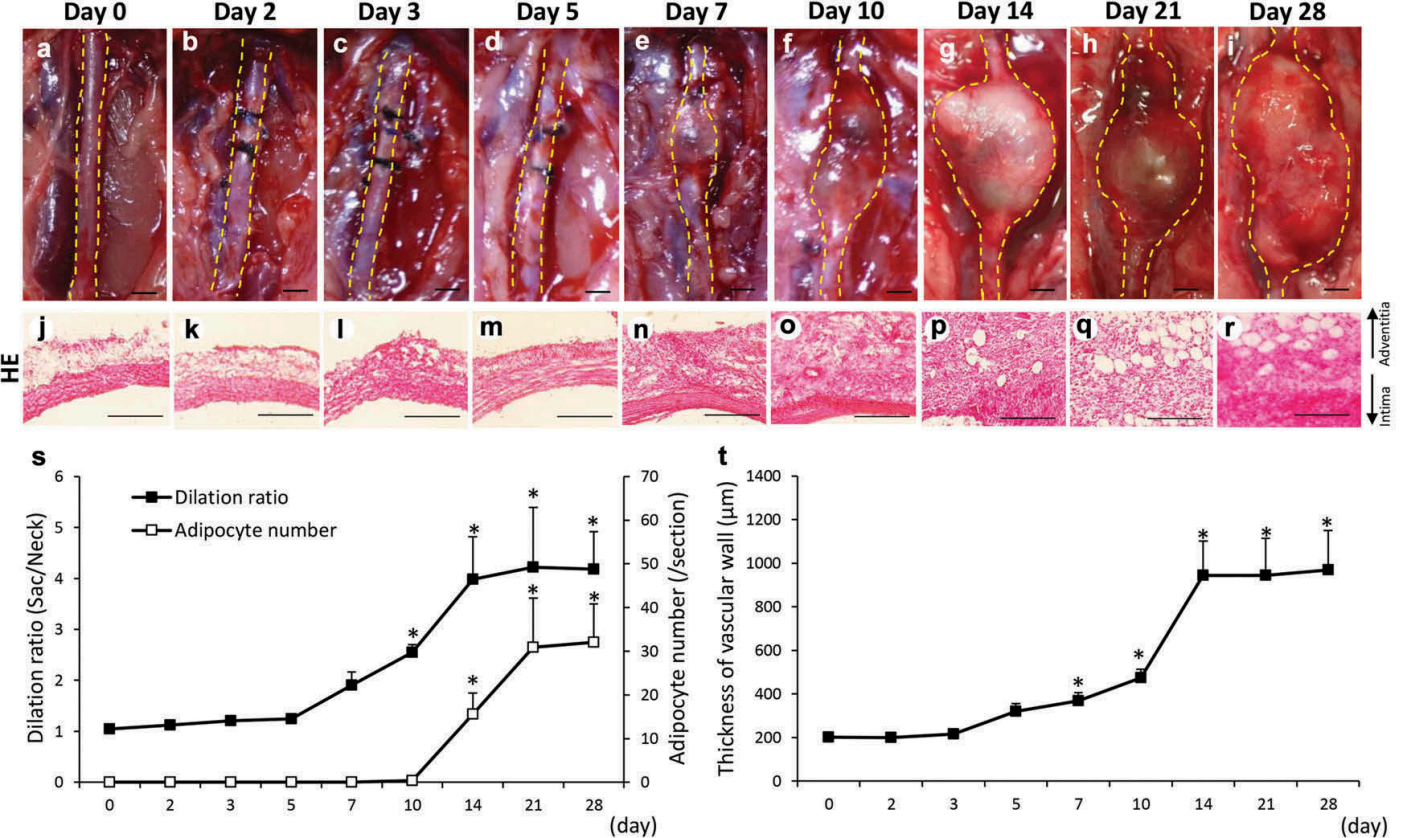
Time-dependent changes of the dilation ratio of the abdominal aorta and the number of adipocytes in the vascular wall. (a–i) Representative images of the abdominal aorta on days 0, 2, 3, 5, 7, 14, 21, and 28 after the induction of hypoperfusion (scale bar = 1.5 mm). (j–r) Representative images of the hematoxylin-eosin (HE) staining (scale bar = 200 µm). (s) Dilation ratio of the abdominal aorta and the number of adipocytes in the vascular wall. (t) Thickness of the vascular wall. Data are expressed as the mean ± SEM. *P < 0.05 versus day 0. Day 0 (n = 5), day 2 (n = 5), day 3 (n = 5), day 5 (n = 5), day 7 (n = 5), day 10 (n = 6), day 14 (n = 5), day 21 (n = 5) and day 28 (n = 7).

Figure 1(a–i) shows the time-dependent change of the abdominal aortae in hypoperfusion-induced animals. The dilation ratio showed a significant increase from 10 to 28 days after the induction of hypoperfusion compared to that on day 0 (Figure 1(s)). The number of adipocytes showed a significant increase from 14 to 28 days after the induction of hypoperfusion compared to that on day 0 (Figure 1(s)). Adipocytes in vascular wall were defined by positively staining with Oil Red O, and the two different adipocyte markers, expressions of peroxisome proliferator-activated receptor γ2 (PPARγ2) and adiponectin (Figure S1). The vascular wall showed significant thickening from 7 to 28 days after the induction of hypoperfusion compared to that on day 0 (Figure 1(j–r, t)). Elastin fibers in the vascular wall were observed using Elastica van Gieson (EVG) staining (Figure S2(a–i)). The elastin degradation score was significantly increased from 5 to 28 days after the induction of hypoperfusion (Figure S2(j)). These results are summarized in Table 1(a) to compare multiple pathological events.

**Table 1.** Time-dependent changes of mesenchymal stem cell (MSC) markers, adipocyte differentiation-related factors, vascular adipocyte number, and abdominal aortic aneurysm (AAA) formation.

	Day 1											
	0 hr	3 hr	6 hr	24 hr	Day 2	Day 3	Day 5	Day 7	Day 10	Day 14	Day 21	Day 28
**(a) Protein expression**												
HIF-1α	-	+	+	+	+	+	+	+	+	+	+	+
CD44	-	-	-	+	+	+	+	+	+	+	+	+
CD90	-	-	-	+	+	+	+	+	+	+	+	+
C/EBPβ	-	-	-	-	-	-	+	+	+	+	+	+
MKL1	-	-	-	-	-	-	-	-	+	+	+	+
PPARγ2	-	-	-	-	-	-	-	-	+	+	+	+
C/EBPα	-	-	-	-	-	-	-	-	-	-	+	+
AAA	-	-	-	-	-	-	-	-	+	+	+	+
Adventitial adipocyte	-	-	-	-	-	-	-	-	-	+	+	+
	Day 0	Day 1	Day 5	Day 10	Day 21							
**(b) mRNA expression**												
HIF-1α	-	+	+	+	+							
CD44	-	+	+	+	+							
CD90	-	-	+	+	+							
C/EBPβ	-	-	+	+	+							
MKL1	-	-	-	+	+							
PPARγ2	-	-	-	+	+							
C/EBPα	-	-	-	+	+							

(a) Time-dependent changes in HIF-1α, MSC markers (CD44 and CD90), adipocyte differentiation-related factors (MKL1, PPARγ2, C/EBPα, and C/EBPβ), number of vascular adipocytes, and AAA formation. ‘+’ indicates P < 0.05 versus day 0 (pre-induction of hypoperfusion). Day 0 (n = 6), day 2 (n = 5), day 3 (n = 5), day 5 (n = 5), day 7 (n = 5), day 10 (n = 5), day 14 (n = 5), day 21 (n = 5), and day 28 (n = 7). (b) Time-dependent changes of mRNA expressions in HIF-1α, MSC markers (CD44 and CD90), adipocyte differentiation-related factors (MKL1, PPARγ2, C/EBPα, and C/EBPβ). Day 0 (n = 6), day 1 (n = 5), day 5 (n = 5), day 10 (n = 5), and day 21 (n = 7).

### Time-dependent changes of hypoxia-inducible factor-1α (HIF-1α) and mesenchymal stem cell (MSC) marker proteins in the hypoperfusion-induced vascular wall

The area positive for HIF-1α was significantly increased from 2 to 28 days after the induction of hypoperfusion (Figure S3(a–j)). HIF-1α mRNA expression significantly increased from 1 to 21 days after the induction of hypoperfusion (Figure S3(k)). The ratio of cells that were positive for the MSC markers CD44^+^ and CD90^+^ showed a significant increase from 2 to 28 days after the induction of hypoperfusion (Figure S4). CD44 mRNA expression significantly increased from 1 to 21 days after the induction of hypoperfusion (Figure S4(t)). CD90 mRNA expression significantly increased from 5 to 21 days after the induction of hypoperfusion (Figure S4(t)). The results for mRNA expression are summarized in Table 1(b) to compare multiple factors. To estimate their expressions within a short-term period, we examined the time-dependent changes for HIF-1α, CD44, and CD90 in the aortic wall from 0 to 24 hours after the induction of hypoperfusion (Figure S5). HIF-1α significantly increased from 3 to 24 hours after the induction of hypoperfusion (Figure S5(m)). CD44 and CD90 significantly increased at 24 hours after the induction of hypoperfusion (Figure S5(m)). Taken together, after the induction of hypoperfusion in the vascular wall, the expression of HIF-1α initially increased followed by an increase in CD44 and CD90 (Table 1).

### Time-dependent changes of adipocyte differentiation-related factors in the hypoperfusion-induced vascular wall

Next, we estimated the time-dependent changes of adipocyte differentiation-related factors. Areas that were positive for megakaryoblastic leukemia 1 (MKL1) and PPARγ2 significantly increased from 10 to 28 days after the induction of hypoperfusion (Figure S6(a–s)). Areas positive for CCAAT/enhancer-binding protein α (C/EBPα) significantly increased from 21 to 28 days after the induction of hypoperfusion (Figure S7(a–i, s)). C/EBPβ significantly increased from 5 to 28 days after the induction of hypoperfusion (Figure S7(j–r, s)). These results are summarized in Table 1(a). MKL1 and PPARγ2 mRNA expressions significantly increased from 10 to 21 days after the induction of hypoperfusion (Table 1(b), Figure S6(t)). C/EBPα mRNA expression significantly increased from 10 to 21 days after the induction of hypoperfusion (Table 1(b), Figure S7(t)). C/EBPβ mRNA expression significantly increased from 5 to 21 days after the induction of hypoperfusion (Table 1(b), Figure S7(t)). In sham operation without aortic wall ligation, AAA was not developed [4,12], and the levels of HIF-1α and PPARγ2 on day 28 were not significantly different compared to those on day 0 (Figure S8).

### Localization of HIF-1α and MSC markers in the hypoperfusion-induced AAA wall

**Figure 2.**
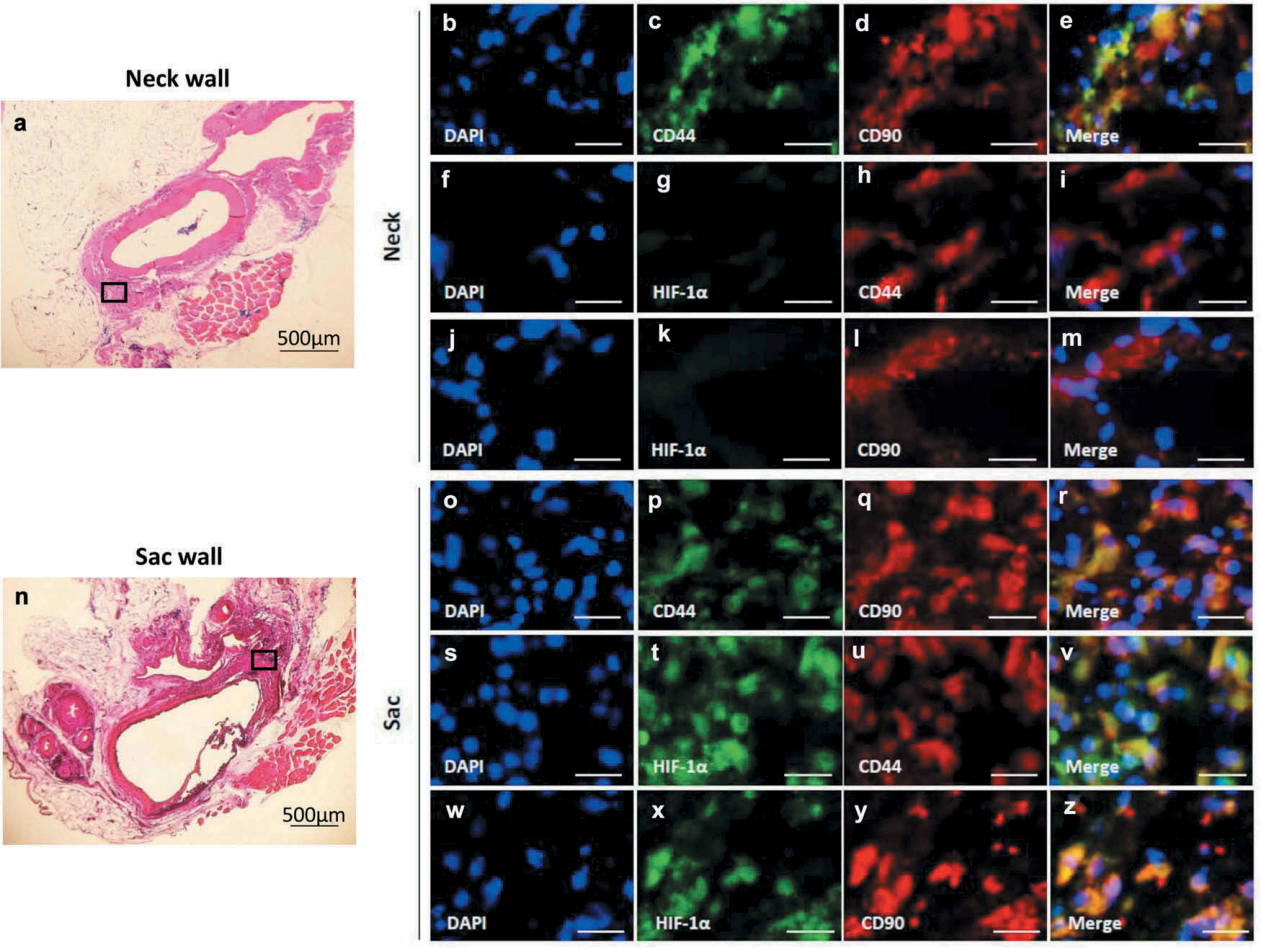
Co-localization of HIF-1α, CD44, and CD90 in the vascular wall. (a) Representative image of the AAA-neck wall with perivascular adipose tissue (PVAT). The squared area is representative area for analysis. Double-immunostaining for CD44 and CD90 (b–e), HIF-1α and CD44 (f–i), and HIF-1α and CD90 (j–m) in the abdominal aortic aneurysm (AAA)-neck wall. (n) Representative image of the AAA-sac wall with PVAT. The squared area is representative area for analysis. Double-immunostaining for CD44 and CD90 (o–r), HIF-1α and CD44 (s–v), and HIF-1α and CD90 (w–z) in the AAA-sac wall. Scale bar = 50 µm. Neck (n = 5) and sac (n = 5) walls.

CD44 co-localized with CD90 in both the AAA-neck (Figure 2(a–e)) and AAA-sac walls (Figure 2(n–r)). CD105 co-localized with CD90, but CD45 did not co-localized with CD90 in both the AAA-neck and AAA-sac walls (Figure S9). HIF-1α co-localized with CD44 and CD90 in the AAA sac wall (Figure 2(s–z)), but not in the AAA-neck wall where hypoperfusion was not induced (Figure 2(f–m)). These data suggest CD44^+^CD90^+^ cells are in a hypoxic condition in the AAA sac wall.

### Localization of adipocyte differentiation-related factors, CD44, and CD90 in the hypoperfusion-induced AAA wall

**Figure 3.**
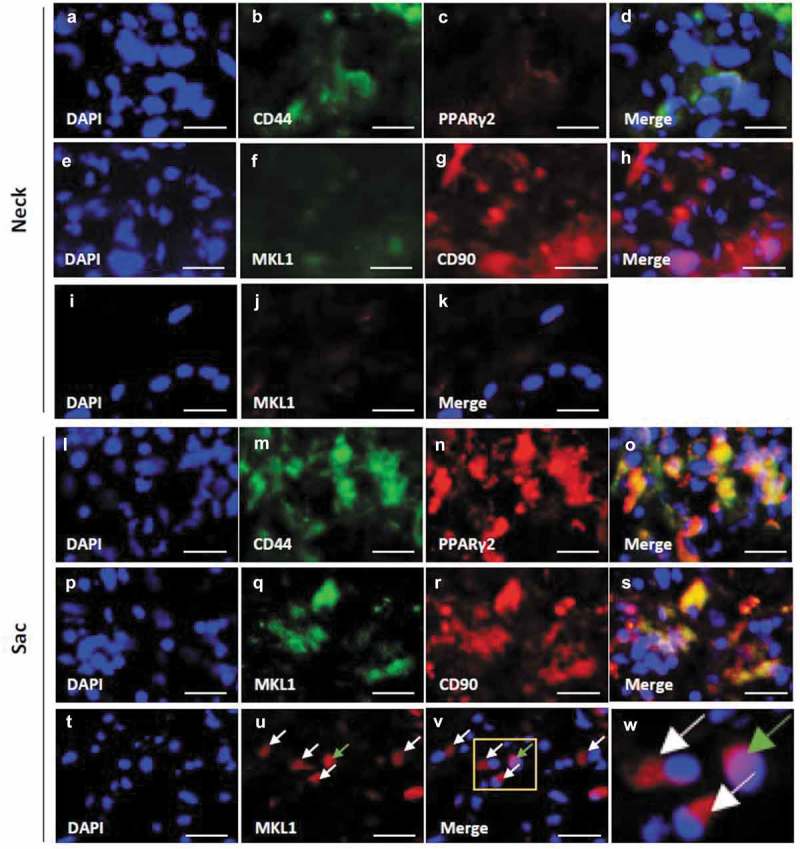
Localization of PPARγ2, MKL1, and mesenchymal stem cell (MSC) markers in the vascular wall. Double-immunostaining for CD44 and PPARγ2 (a–d), MKL1 and CD90 (e–h), and DAPI and MKL1 (i–k) in the abdominal aortic aneurysm (AAA)-neck wall. Double-immunostaining for CD44 and PPARγ2 (l–o), MKL1 and CD90 (p–s), and DAPI and MKL1 (t–v) in the AAA-sac wall. The squared area in the (v) panel is magnified in the (w) panel. The white and green arrows indicate the extranuclear and intranuclear regions, respectively. Scale bar = 50 µm. Neck (n = 5) and sac (n = 5) walls.

Both PPARγ2 and MKL1 were weakly observed in the neck wall (Figure 3(a–h)). PPARγ2 co-localized with CD44 in the AAA-sac wall (Figure 3(l–o)). MKL1 co-localized with CD90 in the AAA-sac wall (Figure 3(p–s)). MKL1 was observed both in the nucleus and extranuclear region in the AAA-sac wall (Figure 3(t–w)). C/EBPα and C/EBPβ were observed in the AAA-sac wall but not in the neck wall (Figure S10(a–h)). C/EBPα and C/EBPβ co-localized with CD90 in the AAA-sac wall (Figure S10(i–p)). These data suggested that CD44^+^ and CD90^+^ cells can differentiate into adipocytes in the AAA wall.

### Removal of perivascular adipose tissue (PVAT) causes a decrease of adipocytes in the hypoperfusion-induced AAA wall

**Figure 4.**
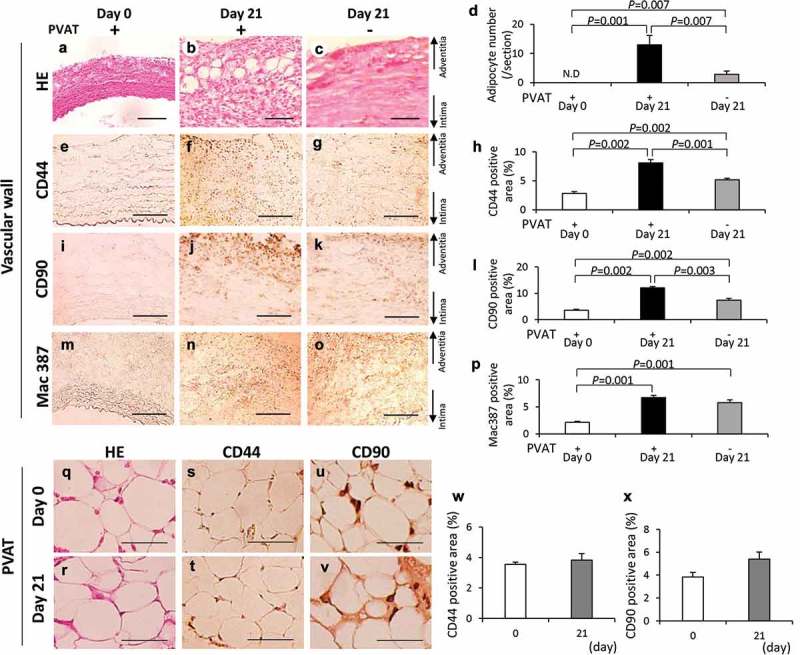
Effects of the perivascular adipose tissue (PVAT) removal on the number of adipocytes and the areas positive for CD44, CD90 and Mac387 in the vascular wall. Representative images of hematoxylin-eosin (HE) staining (a–c) and the number of adipocytes (d). Representative images of the immunostaining for CD44 (e–g) and quantification of CD44 positive areas (h). Representative images of the immunostaining for CD90 (i–k) and quantification of the CD90 positive areas (l). Representative images of the immunostaining for Mac387 monocytes/macrophages (m–o) and quantification of Mac387 positive areas (p). (scale bar = 100 µm) Vascular wall without the induction of hypoperfusion (day 0) (n = 6), vascular wall with the induction of hypoperfusion (day 21) (n = 8) and PVAT-removed vascular wall with the induction of hypoperfusion (day 21) (n = 6). (q, r) Representative images of HE staining of PVAT on days 0 and 21 after the induction of hypoperfusion. Representative images of the immunostaining for CD44 (s, t) and CD90 (u, v). (scale bar = 50 µm) Quantification of areas positive for CD44 (w) and CD90 (x). Day 0 (n = 4) and day 21 (n = 3).

To clarify the effect of removal of PVAT on the ectopic adipocytes, we compared the three kinds of vascular walls: 1, vascular wall without the induction of hypoperfusion (day 0); 2, vascular wall with induction of hypoperfusion (day 21); and 3, PVAT-removed vascular wall with the induction of hypoperfusion (day 21). PVAT was removed when the vascular hypoperfusion was induced (Figure S11). Adipocyte was not detected both in neck wall with and without the removal of PVAT (Figure S12). The number of adipocytes was significantly decreased in the PVAT-removed vascular wall compared with that in the vascular wall with PVAT (Figure 4(a–d)). The positive areas for CD44 were significantly decreased by the removal of PVAT (Figure 4(e–h)). The positive areas for CD90 were significantly decreased by the removal of PVAT (Figure 4(i–l)). The positive areas for Mac 387 in the PVAT-removed vascular wall were not different from that in the vascular wall with PVAT (Figure 4(m–p)). CD44 and CD90 were observed in the PVAT both on days 0 and 21 (Figure 4(q–v)). The PVAT on days 0 and 21 was observed by hematoxylin-eosin (HE) staining (Figure S13). The positive areas for CD44 and CD90 in the PVAT were not significantly different between days 0 and 21 (Figure 4(w, x)). These data suggest that PVAT plays important roles in the appearance of both adipocytes and CD44^+^ and CD90^+^ cells in AAA wall.

### Effect of removal of PVAT on the development of AAA

**Figure 5.**
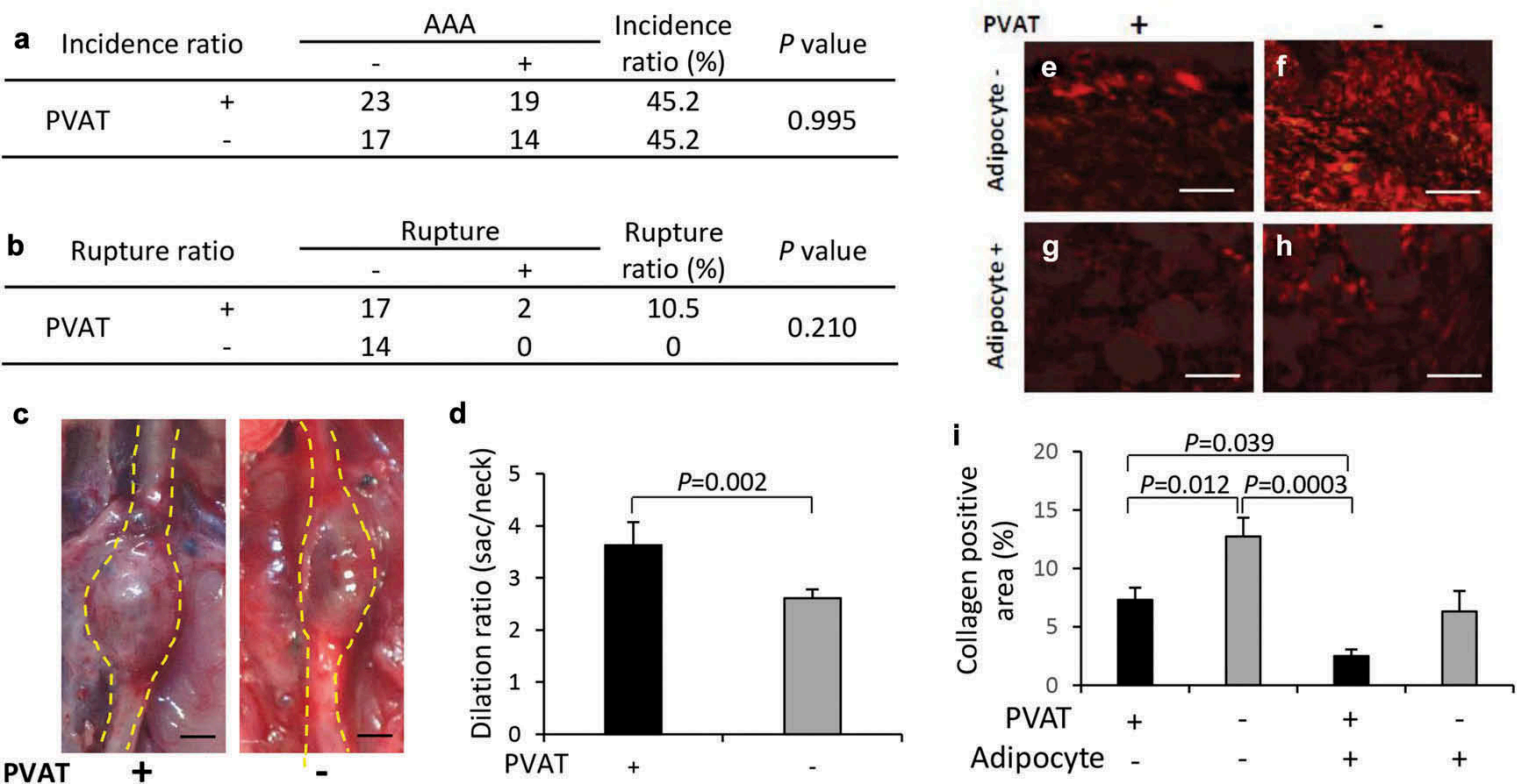
Effect of PVAT removal on the development of AAA. (a) The incidence ratio of AAA (b) The effect of the removal of PVAT on AAA rupture ratio. (c) Representative images of the abdominal aorta on day 21 after the induction of hypoperfusion, and day 21 after the induction of hypoperfusion with the removal of PVAT (scale bar = 1.5 mm). (d) Dilation ratio of the aortic wall. (e-h) Representative images of picrosirius red (PSR) staining (scale bar = 50 µm). (i) Quantification of collagen positive area. The vascular wall with the induction of hypoperfusion (day 21) (n = 19), and PVAT-removed vascular wall with induction of hypoperfusion (day 21) (n = 14).

The incidence rate of AAA was not different between two groups (Figure 5(a)). The AAA diameter (vascular dilation ratio) significantly decreased in the PVAT-removed vascular wall compared with that in the vascular wall with PVAT (Figure 5(b–d)). Collagen-positive areas in the vascular wall significantly increased in the PVAT-removed vascular wall compared with the vascular wall with PVAT in regions without adipocytes (Figure 5(e–i)).

## Discussion

In this study, we performed a time-dependent pathological analysis of the vascular wall of a hypoperfusion-induced AAA model to clarify the molecular mechanism underlying the abnormal appearance of adipocytes in the AAA wall. CD44 and CD90 were co-localized in the aortic wall, and HIF-1α, MKL1, PPARγ2, C/EBPα, and C/EBPβ were co-localized with CD44 and/or CD90. The number of adipocytes and the positive areas for CD44 and CD90 were significantly decreased by day 21 in the PVAT-removed vascular wall. CD44 and CD90 were observed in the PVAT on days 0 and 21. These results suggested that PVAT plays important roles in the differentiation of CD44^+^ and CD90^+^ MSCs into adipocyte in the AAA wall.

MSCs are multipotent stromal cells with self-renewing abilities that can differentiate into a variety of cell types, including adipocytes, chondrocytes, osteoblasts, and smooth muscle cells[13]. C/EBPβ was highly expressed in preadipocytes in the early stages of induction during their differentiation into adipocytes and subsequently, PPARγ expression was induced[14]. During the later stages of differentiation, C/EBPα expression was induced and C/EBPα interacted with PPARγ[14]. Our results were consistent with these processes during adipocyte differentiation. Under hypoxic conditions, human bone marrow-derived MSCs accelerated adipocyte differentiation, and HIF-1α and C/EBPs play important roles in the process of adipocyte differentiation[15]. In the present study, the number of HIF-1α^+^, C/EBPα^+^, and C/EBPβ^+^ cells increased, and these cells co-localized with CD44 and CD90 in the hypoperfusion-induced vascular wall. A recent study reported that actin cytoskeleton dynamics induced adipocyte differentiation[16]. In this study, extranuclear MKL1 expression was observed in CD90^+^ cells in the hypoperfusion-induced vascular walls. Therefore, the increased expression of PPARγ2 observed in the present study may be induced not only via the C/EBP pathway but also via the extranuclear MKL1 pathway. It has been reported that CD44^+^CD90^+^ MSCs were observed in the human AAA wall[17]. Ciavarella et al. reported that MSCs isolated from human AAA tissue can differentiate into several cell types including adipocytes[18]. These data suggest that CD44^+^CD90^+^ MSCs in the AAA wall can differentiate into adipocytes in the vascular wall under hypoxic conditions.

**Figure 6.**
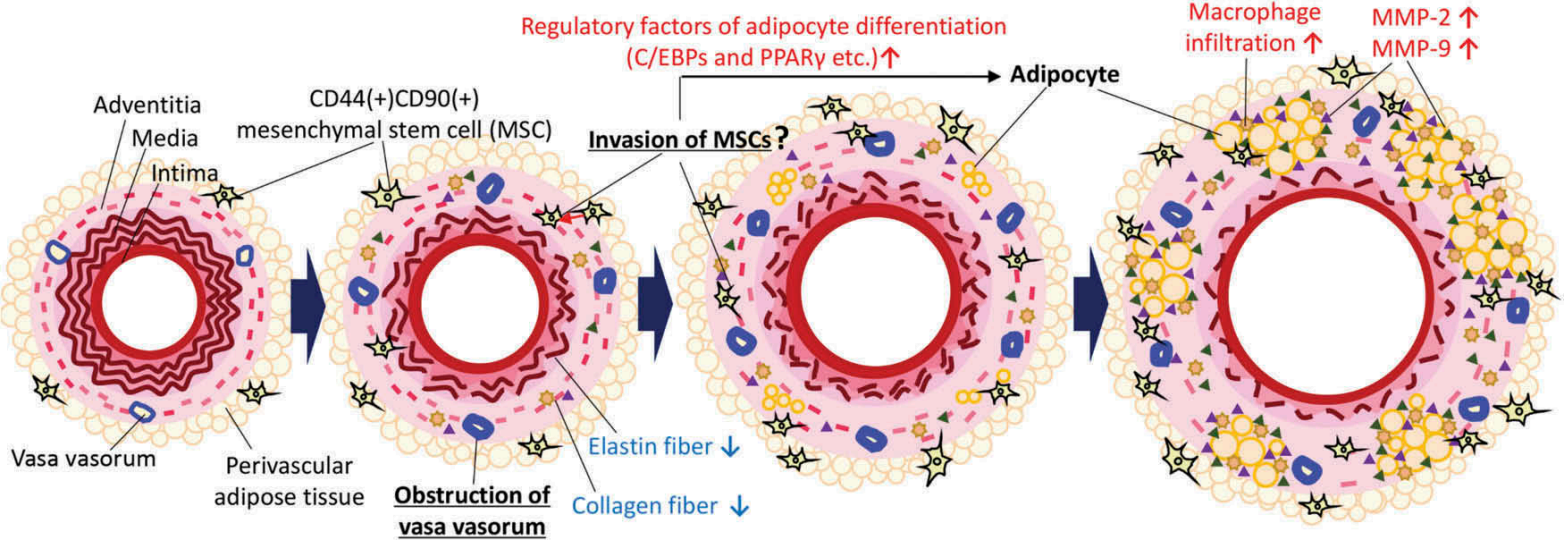
Putative potential mechanism underlying the ectopic appearance of adipocytes in the AAA wall. Hypoperfusion in the aortic wall due to the obstruction of the vasa vasorum resulted in the appearance of CD44^+^CD90^+^ MSCs in the aortic wall. The increased CD44^+^CD90^+^ MSCs differentiated into abnormal adipocytes in the vascular wall with the expression of adipocyte differentiation regulatory factors. The appearance of adipocytes can cause a weakness of the vascular wall. It should be noted that this schema is a simplified focused on the adipocytes in the vascular wall. In the actual AAA wall, various pathological events, not only adipocytes accumulation, but also extracellular matrix degradation, microcalcification, and oxidative stress in the media, are involved in the development of AAA. Further studies are needed to elucidate the relationship between adipocyte infiltration and other AAA events.

A putative potential mechanism underlying the ectopic appearance of adipocytes in the AAA wall is shown in Figure 6. Hypoperfusion due to the stenosis of vasa vasorum [3] induce the migration of MSCs. MSCs contribute to the remodeling of the vascular wall under normal conditions; however, the MSCs express adipocyte differentiation-related factors and differentiated into adipocytes under the abnormal conditions in the AAA wall. Our data suggested that some MSCs in the AAA wall were derived from PVAT. In combination with other pathological events such as inflammation, the increased number of adipocytes in the AAA wall can increase predisposition to rupture of the AAA wall [5,6,12,19,20]. A recent mechanical study suggested that adipocytes are associated with the weakness in the human AAA wall[11], which was consistent with our hypothesis. We speculated that the differentiation from MSCs into adipocytes occurs only under the abnormal malperfusion condition in the AAA wall. Under normal conditions, MSCs might be involved in vascular remodeling by using its multipotent capability. It has been reported that MSCs accumulate at damaged tissues with the involvement of the stromal cell-derived factor 1α/C-X-C type chemokine ligand 4 axis protein under hypoxic conditions[21]. MSCs migrated toward sites of tissue damage in response to high mobility group box 1 protein that is secreted from necrotic cells[22]. Vascular wall-resident MSCs normally differentiate into smooth muscle cells and pericytes to play an important role in the stabilization of the vascular wall [23,24]. Therefore, the use of pluripotent MSCs may be effective for the treatment of AAA. However, our data suggested that the unusual conditions in the AAA wall might disrupt the appropriate control of MSC differentiation. Furthermore, because MSCs in the aortic wall are capable of osteogenic differentiation [18], it has been speculated that MSCs may ectopically differentiate into osteoblasts in the AAA wall under hypoperfused conditions. In addition, it has been reported that MSCs isolated from the human AAA wall significantly increased the expression and activity of MMP-9 compared with healthy MSCs[17]. To establish a therapeutic strategy for AAA, it may be important to consider methods for the mitigation of vascular hypoperfusion or inhibition of abnormal differentiation of MSCs. In this study, we showed the pathological evidence focused on the hypoxic conditions as the result of vascular hypoperfusion. However, hypoperfusion can cause several other impacts on the vascular wall such as malnutrition. Further studies are needed to clarify the gap between hypoperfusion and the abnormal appearance of adipocytes in the AAA wall. Our data showed that positive areas for Mac387 are unaffected by the presence or absence of PVAT, suggesting monocyte/macrophage migrate from luminal blood flow to vascular wall in our experimental model. It is of interest to be able to characterize the origin of other cells in AAA wall in future studies.

Several studies showed that PVAT was involved in the formation of aneurysms in association with inflammatory cell infiltration in PVAT [25–30]. Inflammatory cells were observed in the PVAT–adjacent AAA wall of both human and experimental AAA animal models[31]. Periaortic fat volume was associated with dimensions in the thoracic and abdominal aortae[32]. Dias-Neto et al. reported the intra-individual difference was positively associated with aortic volume[33]. They reported that PVAT density around the aneurysmal sac was higher than that around healthy neck. In this study, the AAA diameter (vascular dilation ratio) significantly decreased in the PVAT-removed vascular wall. It has been reported that the number of adipocytes in the AAA wall correlated with the AAA diameter[5]. The decreased dilation ratio in the PVAT-removed group might be because of the decreased number of adipocytes in the PVAT-removed vascular wall. The weakness/limitation of this study is that we could not conclude the effect of PVAT on AAA rupture due to the limitation of experimental conditions. The rupture ratio in the animal model is usually around 10–15% in normal condition. This low rupture ratio is convenient for time-dependent analysis of the origin of adipocytes in this study. However, it is not suitable for obtaining enough samples to evaluate the effects of PVAT on AAA rupture. We have previously reported that the administration of a high fat diet increased the AAA rupture risk to about 50%[12]. In these conditions, it would be possible to evaluate the effect of PVAT on the rupture risk in a reasonable sample size. Further studies are needed to elucidate the effect of PVAT on the AAA rupture in hypoperfusion-induced AAA animal model.

In conclusion, we show the effect of PVAT on the ectopic vascular adipocytes, which is one of the unrecognized potential contributors of the AAA development. Our data showed the possibility of unreported pathological interactions between PVAT and vascular wall in the development of AAA. The appearance of adipocytes might come as a result of unintended differentiation from MSCs under the vascular hypoperfusion. The maintenance of vasa vasorum blood flow in the vascular wall might be an effective strategy for the prevention of AAA rupture.

## Materials and methods

### Animals

All animal experiments were approved by the Kindai University Animal Care and Use Committee and were performed according to the Kindai University Animal Experimentation Regulations (Approval number: KAAG-25–001). Six-week-old male Sprague-Dawley rats (Japan SLC, Inc., Shizuoka, Japan) were provided with food and water *ad libitum* in a humidity-controlled room, with a 12-hour light and 12-hour dark cycle. The room temperature was maintained at 25 ± 1°C.

After habituation for 1 week, the abdominal aorta was ligated over an inserted catheter in all rats to induce AAA. On days 0, 1, 2, 3, 5, 7, 10, 14, 21, and 28 after the induction of hypoperfusion, aortic diameters were measured, and the rats were sacrificed. All surgery was performed under anesthesia using medetomidine, midazolam, and butorphanol. All efforts were made to minimize suffering.

### Induction of hypoperfusion of the abdominal aortic wall

The induction of hypoperfusion of the abdominal aortic wall was performed as previously described [4–6,34]. First, the infrarenal aorta was exfoliated from the perivascular tissue. In the PVAT-removal treatment group, the PVAT was removed at this point (Figure S11). Next, vessels branching from the abdominal aorta were then ligated with a 5–0 silk suture (Akiyama Seisakusyo Co., Tokyo, Japan) to block the blood supply. A plastic catheter (Medikit, Tokyo, Japan), shortened to 9 mm in length, was inserted via a small incision adjacent to the renal artery branches, and the incision was then repaired with a 6–0 monofilament suture (Alfresa Pharma, Osaka, Japan). The abdominal aorta was ligated with a 5–0 silk suture together with the inserted plastic catheter. Lastly, the 5–0 silk suture that blocked the blood in the aorta was untied and the blood flow was re-initiated. In the sham operation, ligation of the abdominal aorta together with the inserted plastic catheter was not performed.

### Sample collection

The diameter of the abdominal aorta was measured using digital calipers (A&D, Tokyo, Japan). The dilation ratio was calculated according to the following formula: dilation ratio = maximal aneurysm diameter (sac)/non-dilated vascular diameter (neck). In all experimental groups, PVAT was removed from abdominal aorta before isolation. Isolated tissues were fixed in 4% paraformaldehyde (PFA) (Nacalai Tesque, Kyoto, Japan), soaked in sucrose (10, 15, and 20%), and embedded in O.C.T. Compound (Sakura Finetek Japan Co., Ltd.). These tissue samples were then stored at −80°C until use.

### Histological analysis

Isolated aorta cross-sections (10-µm thick) were prepared using a cryostat (CM1850; Leica Microsystems, Wetzlar, Germany) and mounted on glass slides. To compare the sections used for the analysis of time-dependent changes at the same distance from the ligation point, sections within 2 millimeters from the ligation point were prepared and used for staining. Aortic walls were visualized with hematoxylin-eosin (HE), picrosirius red (PSR), Elastica van Gieson (EVG) and immunohistochemical stains. Quantitative analysis of immunohistochemical staining was performed using the ImageJ software (National Institutes of Health, Bethesda, Maryland, USA). The adipocyte number (/section) was counted in 5 to 10 sections per aortic sample. Elastic fibers were categorized into 4 grades: grade 1, intact elastic fibers; grade 2, lack of wave form; grade 3, thinning of the wave form and/or partial disappearance of elastic fibers; and grade 4, complete disappearance of elastic fibers.

### Immunohistochemical staining

PFA-fixed tissue sections were rinsed in phosphate-buffered saline (PBS) with 1% Triton-X100 and then incubated in 10% oxalic acid for 1 hr. For antigen activation, 0.1% trypsin in PBS was added to the tissue sections. Endogenous peroxidases in the tissue sections were blocked using 3% aqueous hydrogen peroxide in methanol for 8 minutes. After washing in PBS, the tissue sections were blocked with Blocking One Histo (Nacalai Tesque, Kyoto, Japan). The sections were then incubated with the appropriate primary antibody overnight at 4°C. The histological results from the aortic wall were assessed after staining using the following antibodies: MSC markers (rabbit anti-CD44 (1:50; Novus Biologicals, Littleton, CO, USA) and goat anti-CD90 (1:100; Santa Cruz Biotechnology, Dallas, TX, USA)), hypoxia-inducible factor (mouse anti-HIF-1α (1:100; Novus Biologicals, Littleton, CO, USA)), and adipocyte differentiation-related factors (rabbit anti-MKL1 (1:50; Bioss Antibodies, Woburn, MA, USA), goat anti-PPARγ2 (1:100; Santa Cruz Biotechnology, Dallas, TX, USA), rabbit anti-C/EBPα (1:50; Santa Cruz Biotechnology, Dallas, TX, USA), mouse anti-C/EBPβ (1:100; Santa Cruz Biotechnology, Dallas, TX, USA), and mouse anti-monocytes/macrophages (Mac387) (1:50; Bio-Rad Laboratories, Hercules, CA, USA)). On the following day, the sections were rinsed in PBS and incubated with the appropriate secondary antibody conjugated to horseradish peroxidase. The slides were developed with DAB (Vector Laboratories, Burlingame, CA, USA), dehydrated in an ethanol series (80, 90, and 100%), cleared in xylene, and covered with a lipid-soluble mounting medium and glass coverslips. Sections of negative control were not subjected to the primary antibodies, and representative images of negative control were shown in Figure S14.

### Immunofluorescence staining

PFA-fixed tissue sections were rinsed in PBS with 1% Triton-X100. For antigen activation, 0.1% trypsin in PBS was added to the tissue sections. After washing in PBS, the tissue sections were blocked with Blocking One Histo. The sections were incubated with the appropriate primary antibody overnight at 4°C. The histological results from the aortic wall were assessed after staining using the following antibodies as described above. CD45 and CD105 in the aortic wall were assessed after staining using the following antibodies: rabbit anti-CD45 (1:50; abcam, Cambridge, UK) and mouse anti-CD105 (1:50; abcam, Cambridge, UK). The antigen was detected with anti-rabbit or anti-mouse Alexa488-, and anti-rabbit or anti-goat Alexa549-conjugated secondary antibody (Rockland Immunochemicals, Limerick, PA, USA). Sections were subjected by DAPI solution (KPL, Gaithersburg, MD, USA) for nuclear staining for 5 minutes. Slides were covered with Fluoromount^TM^ (Diagnostic BioSystems, Pleasanton, CA, USA). Sections of negative control were not subjected to the primary antibodies, and representative images of negative control were shown in Figure S15.

### Real-time PCR

Isolated aortae were homogenized, and total RNA was isolated using Sepasol-RNA II Super (Nacalai Tesque, Kyoto, Japan) and TURBO DNA-free^TM^ Kit (Thermo Fisher Scientific, MA, USA). Synthesis of cDNA was performed with PrimeScript^TM^ II 1^st^ strand cDNA Synthesis Kit (Takara Bio, Shiga, Japan). Real-time PCR was conducted using SYBR Premix Ex Taq II (Takara Bio, Shiga, Japan), and done using TaKaRa PCR thermal cycler (Takara Bio, Shiga, Japan). Primers used for real-time PCR experiments were shown in Table S1. Amplification condition were 5s at 95°C and 30s at 65°C for 40 cycles. Internal control included *Gapdh*.

### Statistical analyses

Values were expressed as mean ± SEM. For between-group comparisons, the Chi-square test was used for categorical variables. Statistical differences were determined by the Steel test, the Steel-Dwass test and the Mann-Whitney U-test. A *P*-value < 0.05 was considered to indicate statistical significance. Statistical analyses were performed using the StatView 5.0 software (SAS Institute, Cary, USA) and the EZR software[35].

## References

[1] Lindsay ME, Dietz HC. Lessons on the pathogenesis of aneurysm from heritable conditions. Nature. 2011;473(7347):308–316. 21593863 10.1038/nature10145 PMC3622871

[2] Aggarwal S, Qamar A, Sharma V, et al Abdominal aortic aneurysm: A comprehensive review. Exp Clin Cardiol. 2011;16(1):11–15. 21523201 PMC3076160

[3] Tanaka H, Zaima N, Sasaki T, et al Adventitial vasa vasorum arteriosclerosis in abdominal aortic aneurysm. PLoS One. 2013;8(2):e57398. 23460850 10.1371/journal.pone.0057398 PMC3583902

[4] Tanaka H, Zaima N, Sasaki T, et al Hypoperfusion of the adventitial vasa vasorum develops an abdominal aortic aneurysm. PLoS One. 2015;10(8):e0134386. 26308526 10.1371/journal.pone.0134386 PMC4550325

[5] Kugo H, Zaima N, Tanaka H, et al Adipocyte in vascular wall can induce the rupture of abdominal aortic aneurysm. Sci Rep. 2016;6:31268. 27499372 10.1038/srep31268 PMC4976321

[6] Kugo H, Zaima N, Tanaka H, et al Pathological analysis of the ruptured vascular wall of hypoperfusion-induced abdominal aortic aneurysm animal model. J Oleo Sci. 2017;66(5):499–506. 28381776 10.5650/jos.ess16219

[7] Tanaka H, Zaima N, Sasaki T, et al Imaging mass spectrometry reveals a unique distribution of triglycerides in the abdominal aortic aneurysmal wall. J Vasc Res. 2015;52(2):127–135. 26345185 10.1159/000439169

[8] Doderer SA, Gabel G, Kokje VBC, et al Adventitial adipogenic degeneration is an unidentified contributor to aortic wall weakening in the abdominal aortic aneurysm. J Vasc Surg. 2017;67(6):1891–1900. 28912007 10.1016/j.jvs.2017.05.088

[9] Gabel G, Northoff BH, Weinzierl I, et al Molecular fingerprint for terminal abdominal aortic aneurysm disease. J Am Heart Assoc. 2017;6(12):e006798. 29191809 10.1161/JAHA.117.006798 PMC5779007

[10] Krueger F, Kappert K, Foryst-Ludwig A, et al AT1-receptor blockade attenuates outward aortic remodeling associated with diet-induced obesity in mice. Clin Sci (Lond). 2017;131(15):1989–2005. 28646121 10.1042/CS20170131

[11] Niestrawska JA, Regitnig P, Viertler C, et al The role of tissue remodeling in mechanics and pathogenesis of abdominal aortic aneurysms. Acta Biomater. 2019;88:149–161. 30735809 10.1016/j.actbio.2019.01.070

[12] Hashimoto K, Kugo H, Tanaka H, et al The effect of a high-fat diet on the development of abdominal aortic aneurysm in a vascular hypoperfusion-induced animal model. J Vasc Res. 2018;55(2):63–74. 29393228 10.1159/000481780

[13] Chamberlain G, Fox J, Ashton B, et al Concise review: mesenchymal stem cells: their phenotype, differentiation capacity, immunological features, and potential for homing. Stem Cells. 2007;25(11):2739–2749. 17656645 10.1634/stemcells.2007-0197

[14] Rosen ED, MacDougald OA Adipocyte differentiation from the inside out. Nat Rev Mol Cell Biol. 2006;7(12):885–896. 17139329 10.1038/nrm2066

[15] Jiang C, Sun J, Dai Y, et al HIF-1A and C/EBPs transcriptionally regulate adipogenic differentiation of bone marrow-derived MSCs in hypoxia. Stem Cell Res Ther. 2015;6:21. 25889814 10.1186/s13287-015-0014-4 PMC4559195

[16] Nobusue H, Onishi N, Shimizu T, et al Regulation of MKL1 via actin cytoskeleton dynamics drives adipocyte differentiation. Nat Commun. 2014;5:3368. 24569594 10.1038/ncomms4368

[17] Ciavarella C, Alviano F, Gallitto E, et al Human vascular wall mesenchymal stromal cells contribute to abdominal aortic aneurysm pathogenesis through an impaired immunomodulatory activity and increased levels of matrix metalloproteinase-9. Circ J. 2015;79(7):1460–1469. 25854712 10.1253/circj.CJ-14-0857

[18] Ciavarella C, Gallitto E, Ricci F, et al The crosstalk between vascular MSCs and inflammatory mediators determines the pro-calcific remodelling of human atherosclerotic aneurysm. Stem Cell Res Ther. 2017;8(1):99. 28446225 10.1186/s13287-017-0554-x PMC5406974

[19] Kugo H, Moriyama T, Adipocytes ZN Abdominal aortic aneurysm: putative potential role of adipocytes in the process of AAA development. Curr Drug Targets. 2018;19(11):1228–1232. 29336260 10.2174/1389450119666180115164103

[20] Kugo H, Tanaka H, Moriyama T, et al Pathological implication of adipocytes in AAA development and the rupture. Ann Vasc Dis. 2018;11(2):159–168. 30116407 10.3400/avd.ra.17-00130 PMC6094042

[21] Iinuma S, Aikawa E, Tamai K, et al Transplanted bone marrow-derived circulating PDGFRalpha+ cells restore type VII collagen in recessive dystrophic epidermolysis bullosa mouse skin graft. J Immunol. 2015;194(4):1996–2003. 25601922 10.4049/jimmunol.1400914 PMC4319308

[22] Palumbo R, Galvez BG, Pusterla T, et al Cells migrating to sites of tissue damage in response to the danger signal HMGB1 require NF-kappaB activation. J Cell Biol. 2007;179(1):33–40. 17923528 10.1083/jcb.200704015 PMC2064729

[23] Klein D, Weisshardt P, Kleff V, et al Vascular wall-resident CD44+ multipotent stem cells give rise to pericytes and smooth muscle cells and contribute to new vessel maturation. PLoS One. 2011;6(5):e20540. 21637782 10.1371/journal.pone.0020540 PMC3102739

[24] Klein D Vascular wall-resident multipotent stem cells of mesenchymal nature within the process of vascular remodeling: cellular basis, clinical relevance, and implications for stem cell therapy. Stem Cells Int. 2016;2016:1905846. 26880936 10.1155/2016/1905846 PMC4736960

[25] Police SB, Thatcher SE, Charnigo R, et al Obesity promotes inflammation in periaortic adipose tissue and angiotensin II-induced abdominal aortic aneurysm formation. Arterioscler Thromb Vasc Biol. 2009;29(10):1458–1464. 19608970 10.1161/ATVBAHA.109.192658 PMC2753598

[26] Kurobe H, Hirata Y, Matsuoka Y, et al Protective effects of selective mineralocorticoid receptor antagonist against aortic aneurysm progression in a novel murine model. J Surg Res. 2013;185(1):455–462. 23731681 10.1016/j.jss.2013.05.002

[27] Li MW, Mian MO, Barhoumi T, et al Endothelin-1 overexpression exacerbates atherosclerosis and induces aortic aneurysms in apolipoprotein E knockout mice. Arterioscler Thromb Vasc Biol. 2013;33(10):2306–2315. 23887640 10.1161/ATVBAHA.113.302028

[28] Blomkalns AL, Gavrila D, Thomas M, et al CD14 directs adventitial macrophage precursor recruitment: role in early abdominal aortic aneurysm formation. J Am Heart Assoc. 2013;2(2):e000065. 23537804 10.1161/JAHA.112.000065 PMC3647288

[29] Sakaue T, Suzuki J, Hamaguchi M, et al Perivascular adipose tissue angiotensin II type 1 receptor promotes vascular inflammation and aneurysm formation. Hypertension. 2017;70(4):780–789. 28760942 10.1161/HYPERTENSIONAHA.117.09512

[30] Zhang ZB, Ruan CC, Lin JR, et al Perivascular adipose tissue-derived PDGF-D contributes to aortic aneurysm formation during obesity. Diabetes. 2018;67(8):1549–1560. 29794241 10.2337/db18-0098

[31] Folkesson M, Vorkapic E, Gulbins E, et al Inflammatory cells, ceramides, and expression of proteases in perivascular adipose tissue adjacent to human abdominal aortic aneurysms. J Vasc Surg. 2017;65(4):1171–1179 e1171. 26960947 10.1016/j.jvs.2015.12.056

[32] Thanassoulis G, Massaro JM, Corsini E, et al Periaortic adipose tissue and aortic dimensions in the Framingham Heart Study. J Am Heart Assoc. 2012;1(6):e000885. 23316310 10.1161/JAHA.112.000885 PMC3540669

[33] Dias-Neto M, Meekel JP, van Schaik TG, et al High density of periaortic adipose tissue in abdominal aortic aneurysm. Eur J Vasc Endovasc Surg. 2018;56(5):663–671. 30115505 10.1016/j.ejvs.2018.07.008

[34] Kugo H, Zaima N, Mouri Y, et al The preventive effect of fish oil on abdominal aortic aneurysm development. Biosci Biotechnol Biochem. 2016;80(6):1186–1191. 27022887 10.1080/09168451.2016.1146073

[35] Kanda Y Investigation of the freely available easy-to-use software ‘EZR’ for medical statistics. Bone Marrow Transplant. 2013;48(3):452–458. 23208313 10.1038/bmt.2012.244 PMC3590441

