# Supplementary material for: The role of perivascular adipose tissue in the appearance of ectopic adipocytes in the abdominal aortic aneurysmal wall
Source: Adipocyte. 2019 Jun 28;8(1):229–39. doi: 10.1080/21623945.2019.1636625 (PMC6768265; doi:10.1080/21623945.2019.1636625)
Supplement: Supplemental Material [file kadi-08-01-1636625-s001.pdf]

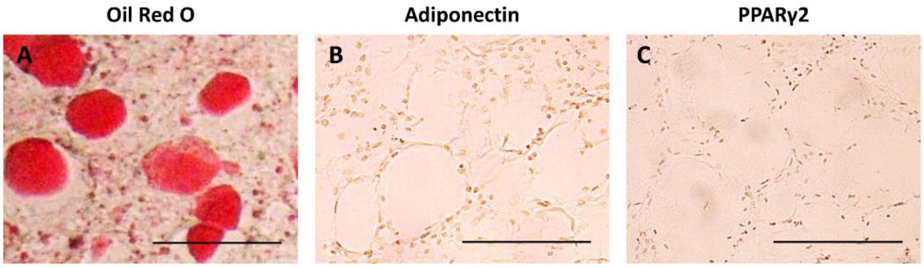

Figure S1. Immunohistochemical staining for PPARγ2 and adiponectin in vascular adipocyte. Representative images of the Oil Red O staining (**A**) and the immunostaining for adiponectin (**B**) and PPARγ2 (**C**) (scale bar = 100 μm).

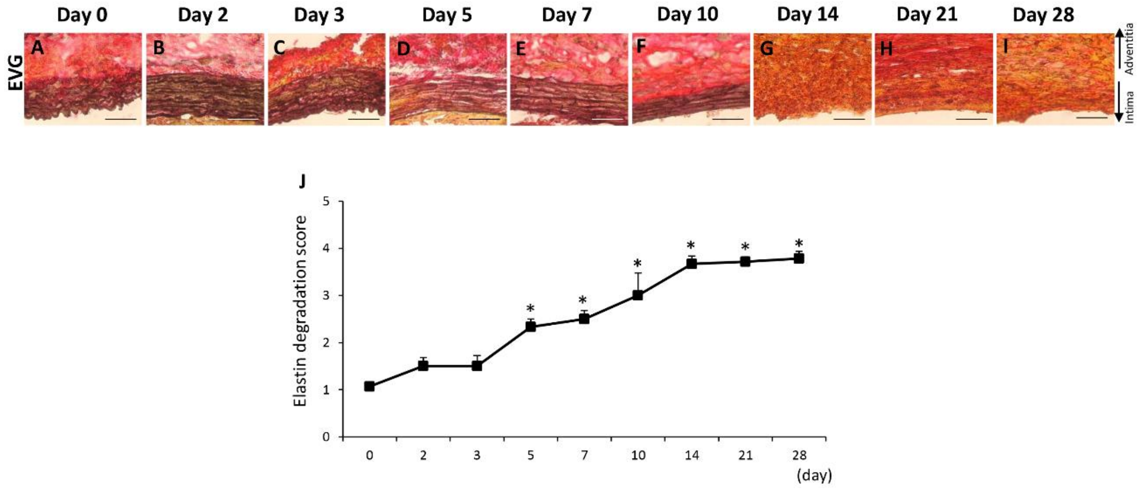

Figure S2. Time-dependent changes of elastin degradation score. ***(*****A-I**) Representative images of the Elastica van Gieson (EVG) staining (scale bar = 50 μm). (**J**) Elastin degradation score. Data are expressed as the mean ± SEM. **P* < 0.05 versus day 0. Day 0 (n = 7), day 2 (n = 6), day 3 (n = 5), day 5 (n = 6), day 7 (n = 6), day 10 (n = 5), day 14 (n = 6), day 21 (n = 14) and day 28 (n = 7).

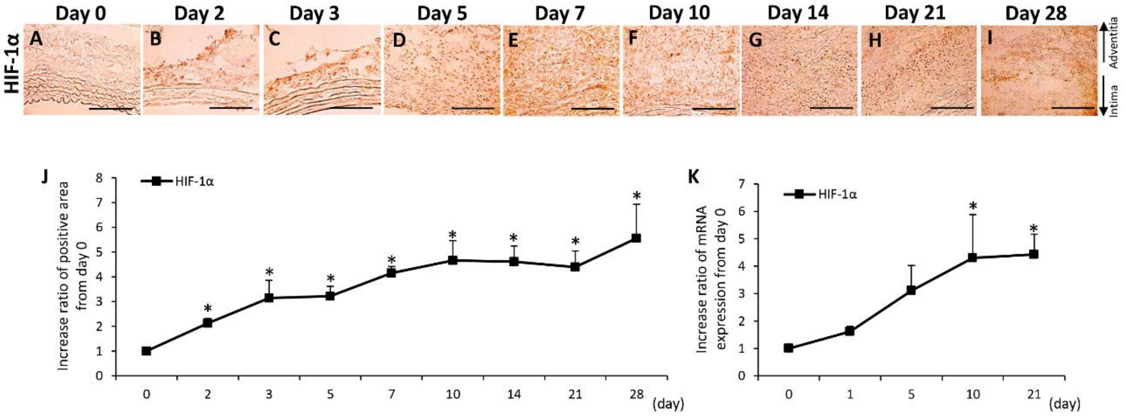

Figure S3. Time-dependent changes of HIF-1α from day 0 to 28. (**A-I**) Representative images of the immunostaining for HIF-1α (scale bar = 100 μm). (**J**) Quantification of the ratio of increase from day 0 of the area positive for HIF-1α in the vascular wall. Day 0 (n = 6), day 2 (n = 5), day 3 (n = 5), day 5 (n = 5), day 7 (n = 5), day 10 (n = 5), day 14 (n = 5), day 21 (n = 8) and day 28 (n = 7). Data are expressed as the mean ± SEM. **P* < 0.05 versus day 0.

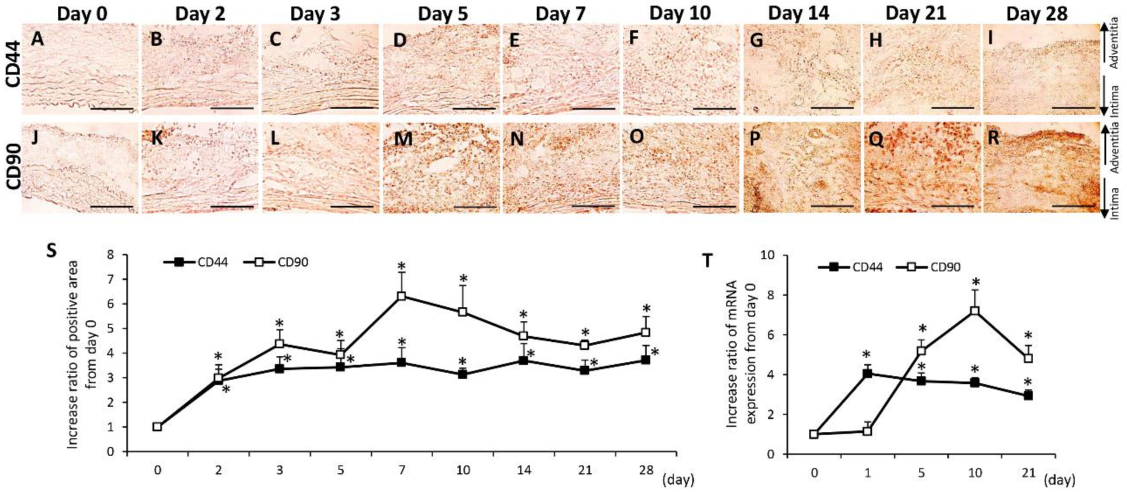

Figure S4. Time-dependent changes of CD44 and CD90 from days 0 to 28. (**A-I**) Representative images of the immunostaining for CD44 (scale bar = 100 μm). (**J-R**) Representative images of the immunostaining for CD90 (scale bar = 100 μm). (**S**) Quantification of the ratio of increase from day 0 of areas positive for CD44 and CD90 in the vascular wall. Data are expressed as the mean ± SEM. **P* < 0.05 versus day 0. Day 0 (n = 6), day 2 (n = 5), day 3 (n = 5), day 5 (n = 5), day 7 (n = 5), day 10 (n = 5), day 14 (n = 5), day 21 (n = 8), and day 28 (n = 7).

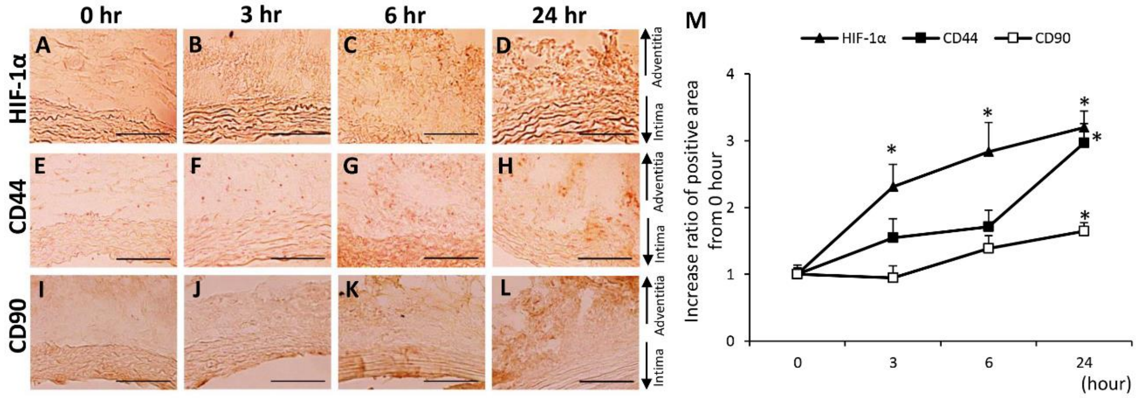

Figure S**5****. Time-dependent changes of HIF-1α, CD44, and CD90 from 0 to 24 h.** (**A-D**) Representative images of the immunostaining for HIF-1α (scale bar = 100 μm). (**E-H**) Representative images of the immunostaining for CD44 (scale bar = 100 μm). (**J-L**) Representative images of the immunostaining for CD90 (scale bar = 100 μm). (**M**) Quantification of the ratio of increase from 0 h of the areas positive for HIF-1α, CD44, and CD90 in the vascular wall. Data are expressed as the mean ± SEM. **P* < 0.05 versus 0 hr. 0 hr (n = 5), 3 hr (n = 5), 6 hr (n = 5), and 24 hr (n = 5).

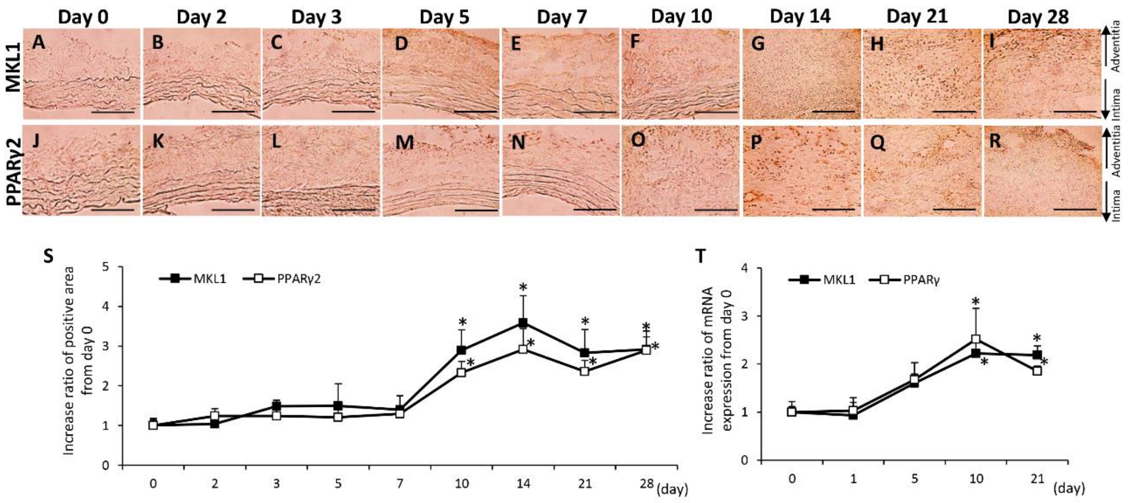

Figure S**6****. Time-dependent changes of MKL1 and PPARγ2.** (**A-I**) Representative images of the immunostaining for MKL1 (scale bar = 100 μm). (**J-R**) Representative images of the immunostaining for PPARγ2 (scale bar = 100 μm). (**S**) Quantification of the ratio of increase from day 0 of areas positive for MKL1 and PPARγ2 in the vascular wall. Day 0 (n = 6), day 2 (n = 5), day 3 (n = 5), day 5 (n = 5), day 7 (n = 5), day 10 (n = 5), day 14 (n = 5), day 21 (n = 8), and day 28 (n = 7). Data are expressed as the mean ± SEM. **P* < 0.05 versus day 0.

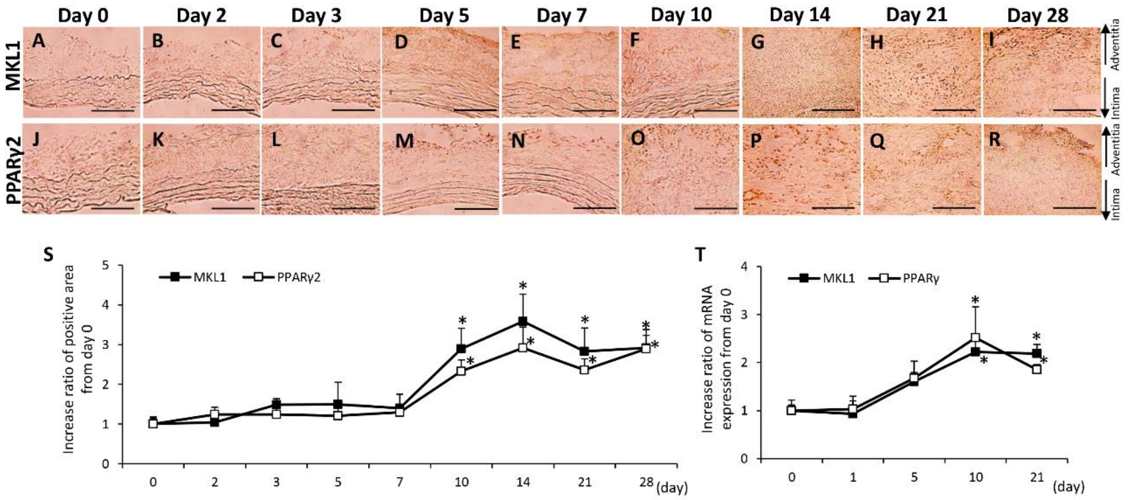

Figure S7. Time-dependent changes of C/EBPα and C/EBPβ. (**A-I**) Representative images of the immunostaining for C/EBPα (scale bar = 100 μm). (**J-R**) Representative images of the immunostaining for C/EBPβ (scale bar = 100 μm). (**S**) Quantification of the ratio of increase from day 0 of the areas positive for C/EBPα and C/EBPβ in the vascular wall. Day 0 (n = 6), day 2 (n = 5), day 3 (n = 5), day 5 (n = 5), day 7 (n = 5), day 10 (n = 5), day 14 (n = 5), day 21 (n = 8) and day 28 (n = 7). Data are expressed as the mean ± SEM. **P* < 0.05 versus day 0.

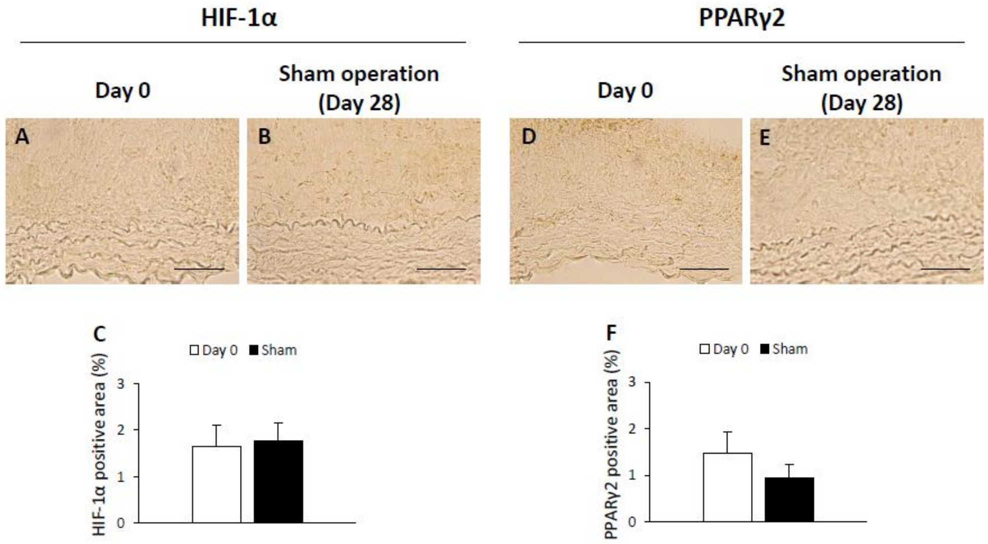

Figure S8. Immunohistochemical staining for PPARγ2 and HIF-1α in the sham-operated aortic wall. (**A, B**) Representative images of the immunostaining for HIF-1α (Scale bar = 50 μm). (**C**) Quantification of the HIF-1α positive area. (**D, E**) Representative images of the immunostaining for PPARγ2 (Scale bar = 50 μm). (**F**) Quantification of the PPARγ2 positive area. Day 0 (n = 5) and sham operation (n = 5). Data are expressed as the mean ± SEM.

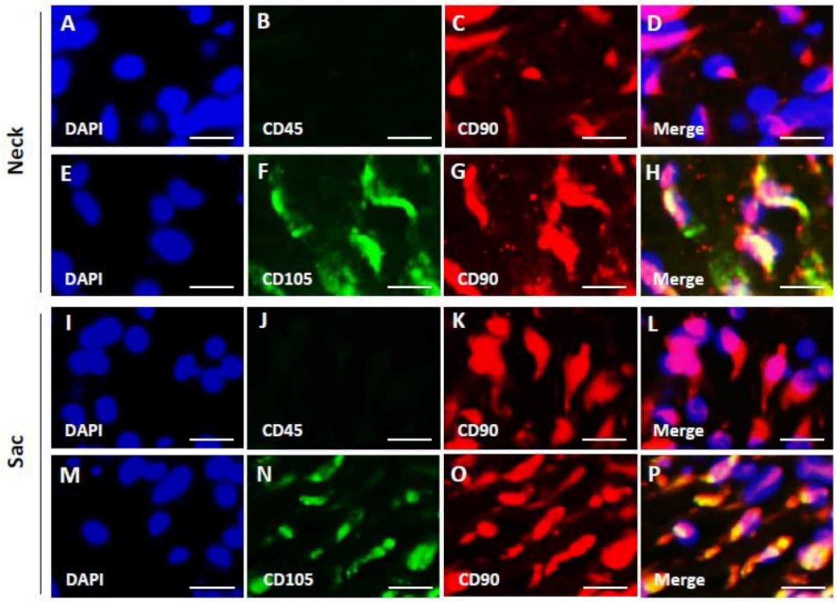

Figure S9. Co-localization of CD105 and CD90 in the vascular wall. Double-immunostaining for CD45 and CD90 (**A-D**), and CD105 and CD90 (**E-H**) in the abdominal aortic aneurysm (AAA)-neck wall. Double-immunostaining for CD45 and CD90 (**I-L**), and CD105 and CD90 (**M-P**) in the AAA-sac wall. Scale bar = 50 µm. Neck (n = 5) and sac (n = 5) walls.

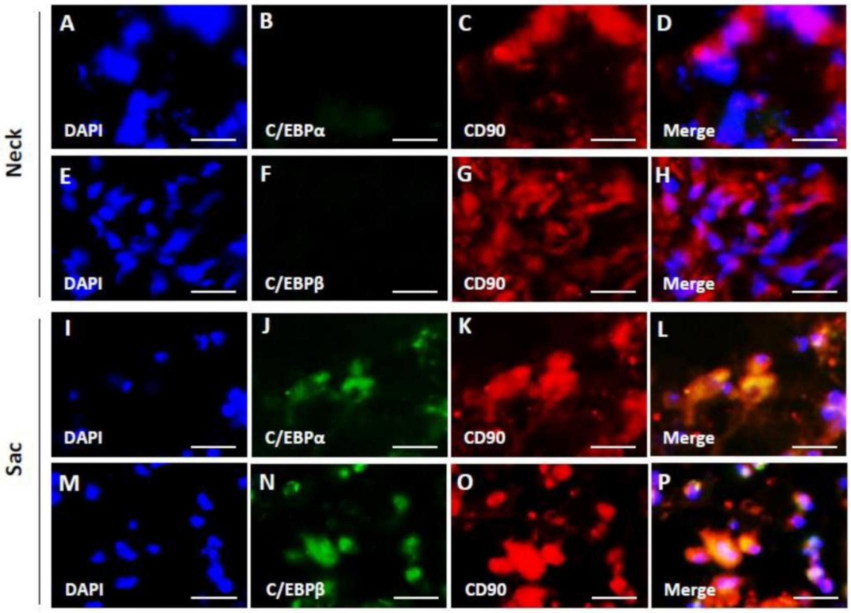

Figure S10. Co-localization of C/EBPα, C/EBPβ, and CD90 in the vascular wall. Double-immunostaining for C/EBPα and CD90 (**A-D**), and C/EBPβ and CD90 (**E-H**) in the abdominal aortic aneurysm (AAA)-neck wall. Double-immunostaining for C/EBPα and CD90 (**I-L**), and C/EBPβ and CD90 (**M-P**) in the AAA-sac wall. Scale bar = 50 µm. Neck (n = 5) and sac (n = 5) walls.

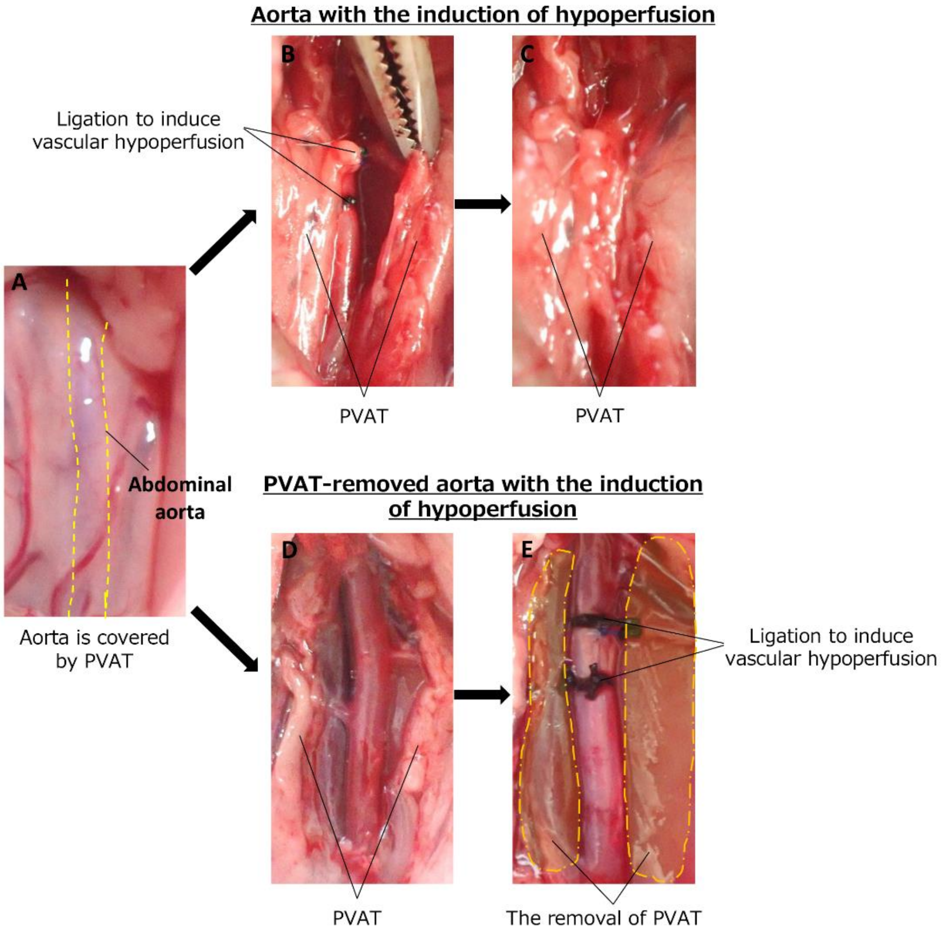

Figure S11. Induction of the abdominal aortic wall with the removal of perivascular adipose tissue (PVAT). (**A**) Abdominal aorta is normally covered by PVAT. (**B, C**) In the treatment without the removal of PVAT, the abdomen was closured in the state of the presence of PVAT after the induction of hypoperfusion in aortic wall. (**D, E**) In the treatment with the removal of PVAT, the abdomen was closured in the state of the absence of PVAT after the induction of hypoperfusion in aortic wall.

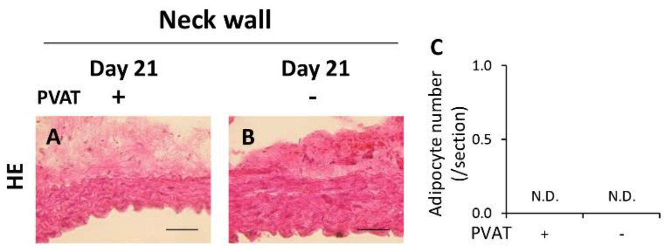

Figure S12. Observation of adipocytes in AAA neck wall. Representative images of hematoxylin-eosin (HE) staining (**A, B**) and the number of adipocytes (**C**) in the AAA neck wall (scale bar = 50 μm).

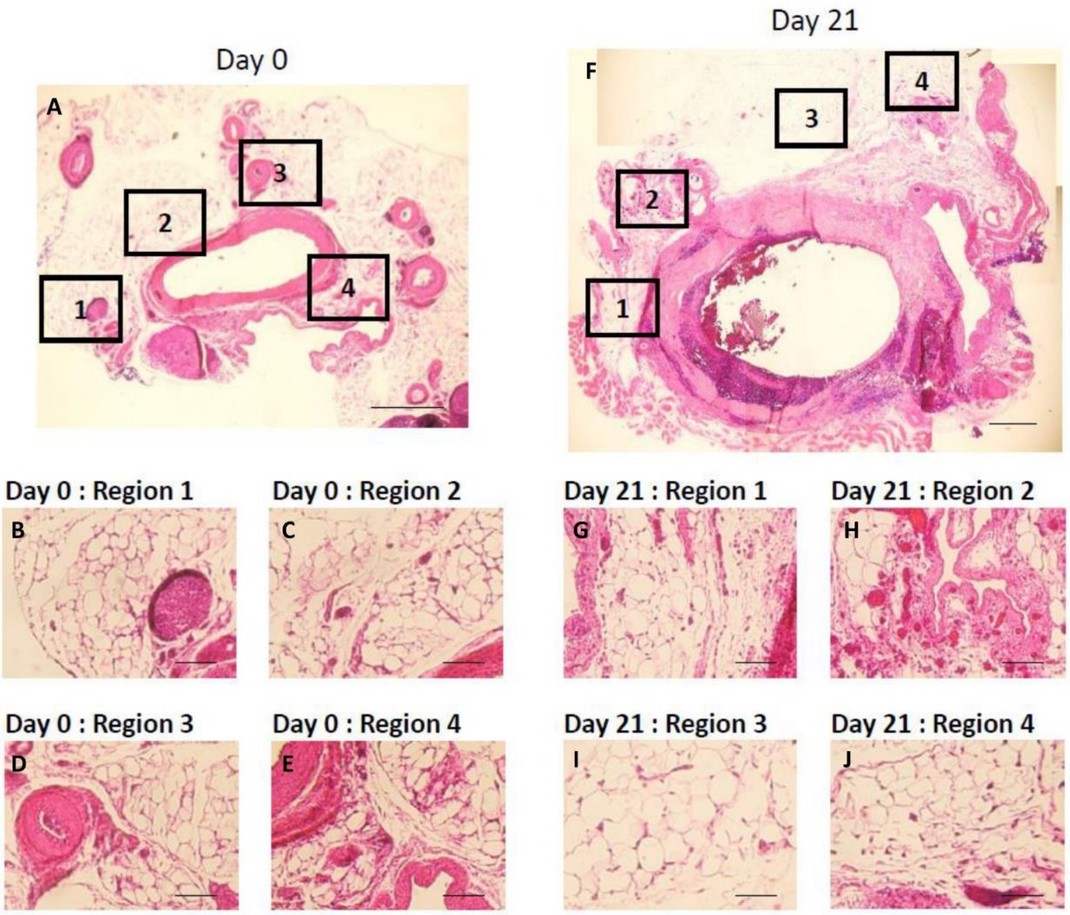

Figure S13. Observation of adipocytes in the perivascular adipose tissue (PVAT). Representative images of the PVAT and aortic wall on day 0 (**A-E**) and day 21 (**F-J**) (A, F: scale bar = 500 µm, B-E, G-J: 100 µm).

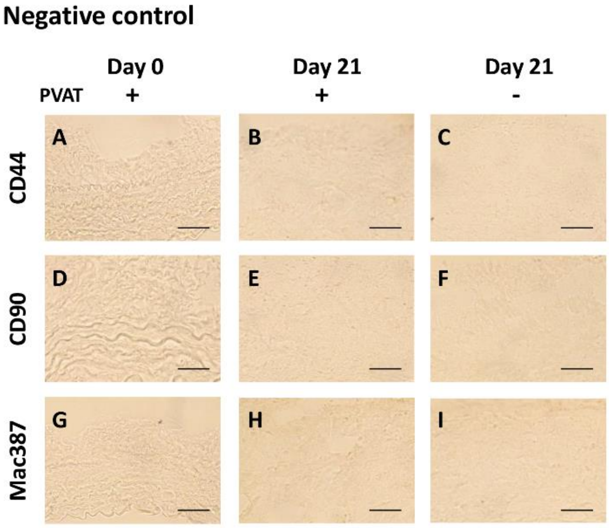

Figure S14. Negative controls of immunohistochemical staining. Representative images of negative stain controls of CD44 (**A-C**), CD90 (**D-F**) and Mac387 (**G-I**). Scale bar = 50 μm.

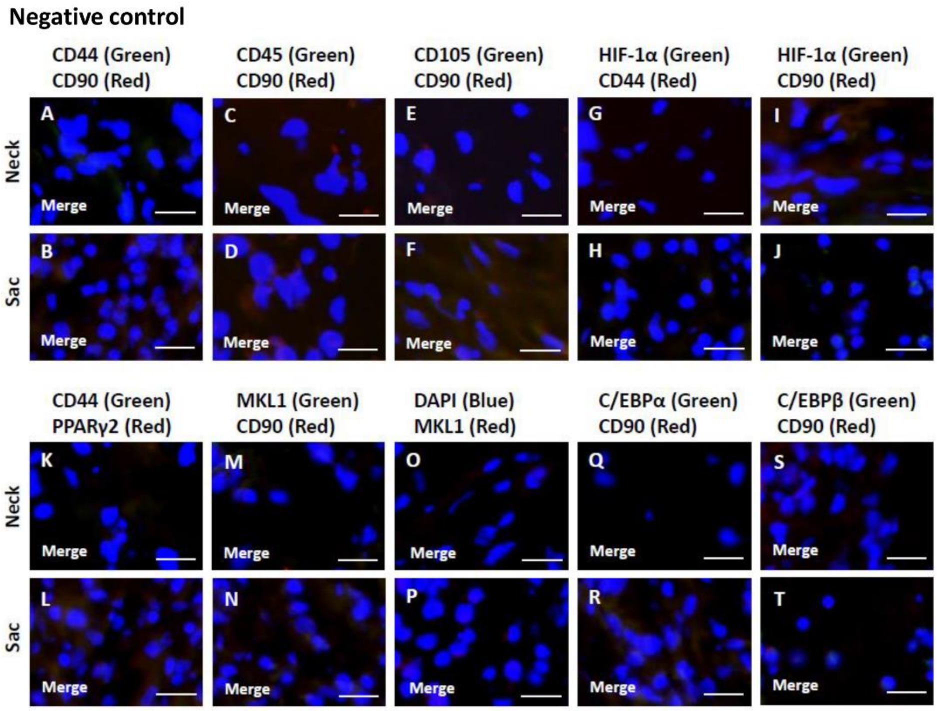

Figure S15. Negative controls of immunofluorescence staining. Representative images of negative stain controls of CD44 and CD90 (**A, B**), CD45 and CD90 (**C, D**), CD105 and CD90 (**E, F**), HIF-1α and CD44 (**G, H**), HIF-1α and CD90 (**I, J**), CD44 and PPARγ2 (K, L), MKL1 and CD90 (**M, N**), DAPI and MKL1 (**O, P**), C/EBPα and CD90 (**Q, R**) and C/EBPβ and CD90 (**S, T**) in the neck and the sac walls. Nuclear staining was shown by blue-fluorescent DAPI. Scale bar = 50 μm.

Table S1. Primers used for real-time PCR experiments.

Gene		Primer sequence
HIF-1α	FWD	5'-TACTGCAGCAACCAGGTGAC-3'
	REV	5'-CTGCCTTGTATGGGAGCATT-3'
CD44	FWD	5'-TGGATCCGAATTAGCTGGAC-3'
	REV	5'-GCTTCTTCTTCTGCCCACAC-3'
CD90	FWD	5'-GAGGGCGACTACATGTGTGA-3'
	REV	5'-AAGGAGAGGGAAAGCAGGAG-3'
C/EBPα	FWD	5'-TTACAACAGGCCAGGTTTCC-3'
	REV	5'-CAGTACACACAAGGCGGATG-3'
C/EBPβ	FWD	5'-GTTTCGGGACTTGATGCAAT-3'
	REV	5'-CAAAAACAAAAACATCAACAGCA-3'
PPARγ2	FWD	5'-TCATGACCAGGGAGTTCCTC-3'
	REV	5'-GGCGGTCTCCACTGAGAATA-3'
MKL1	FWD	5'-CAGCACATGGATGATCTGTTTGA-3'
	REV	5'-GCCCATATGCTTCTGCTGGA-3'
GAPDH	FWD	5'-AAACCCATCACCATCTTCCA-3'
	REV	5'-GTGTTCACACCCATCACAA-3'
